# Improving Staff Knowledge and Confidence in Lithium Counselling to Enhance Patient Safety and Standardise Practice Across Community Mental Health Teams

**DOI:** 10.1192/bjo.2025.10340

**Published:** 2025-06-20

**Authors:** Ella Bauwens, Nazim Zaim, Yousof Osman, Khyati Patel, Nirvana Islam

**Affiliations:** 1 King’s College London, London, United Kingdom.; 2 KMPT, Kent, United Kingdom

## Abstract

**Aims:** The 2019–20 Community Pharmacy Quality Scheme audit concluded 34% of audited patients were unfamiliar with lithium toxicity symptoms, where 29% were unaware of how to prevent it, highlighting the gap in effective patient education. Our preliminary research revealed that 50% of medical professionals lacked confidence in providing lithium counselling with 41% being either unaware or unsure of how to counsel a patient if they had missed a dose. Therefore, we aim to tackle staff knowledge and improve abilities in lithium counselling to enhance patient safety and understanding, ultimately leading to fewer incidences of toxicity and harm.

**Methods:** An initial survey was conducted to assess healthcare professionals’ confidence in lithium counselling prior to the teaching sessions, identifying specific gaps in knowledge among staff. The Quality Improvement Project was implemented through two Plan-Do-Study-Act (PDSA) cycles:

PDSA Cycle 1 (19 attendees): A lecture-based teaching session using an online presentation was delivered, covering key information regarding lithium counselling. An improvement in knowledge was assessed using pre- and post-session quizzes, created using the information in the “Lithium Policy KMPT Handbook”.

PDSA Cycle 2 (6 attendees): An interactive OSCE-based teaching session was delivered to reinforce and apply the content from PDSA 1 via two clinical-based scenarios including discussion and feedback.

All teaching material was distributed to staff members, and the session was recorded for future training opportunities, accounting for standardised teaching methods.

**Results:** The baseline average score was 50%. Following the PDSA 1 session, this increased to 79%, demonstrating a statistically significant improvement (t = ×5.14, p<0.001). Following the PDSA 2 session, there was a slight decrease to 77%.

Key areas of knowledge that showed notable improvement after PDSA 1 included:

1 – Missed Dose Advice: χ² (1, N=37 =0.000154, p=0.05.

2 – Lithium use in pregnancy: χ² (1, N=37)=0.00056, p=0.05.

3 – Initiating lithium monitoring: χ² (1, N=37)=0.004238, p=0.05.

Direct comparison between post PDSA 1 and 2 is limited due to the lack of participant continuity.

**Conclusion:** After PDSA 1, a clear improvement in staff scores was observed. Despite showing a slight decrease in knowledge after PDSA 2, both teaching methods proved effective in improving lithium counselling knowledge from baseline.

We hypothesise that attending both sessions would lead to the greatest improvement; however, scheduling constraints prevented consistent attendance. Attempting to account for this, sessions were planned online. Upon reflection, recording and disseminating all teaching resources were vital in ensuring standardised training.

